# Effectiveness of Borage plus syrup on COVID-19 patients in intensive care units

**DOI:** 10.3389/fnut.2022.975937

**Published:** 2022-11-15

**Authors:** Seyed MohammadReza Hashemian, Esmaeil Mortaz, Navid Shafigh, Shadi Ziaie, Hamidreza Jamaati, Morteza Hasheminik, Mehdi Jamalinik, Raziyeh Erfani, Batoul Khoundabi, Neda K. Dezfuli, Mohammad Varahram, Shahrzad Ahmadi, Mahdi Fahimi, Ian M. Adcock

**Affiliations:** ^1^Chronic Respiratory Diseases Research Center, National Research Institute of Tuberculosis and Lung Diseases (NRITLD), Shahid Beheshti University of Medical Sciences, Tehran, Iran; ^2^Department of Anesthesiology and Critical Care Medicine, School of Medicine, Shahid Beheshti University of Medical Sciences, Tehran, Iran; ^3^Department of Clinical Pharmacy, School of Pharmacy, Shahid Beheshti University of Medical Sciences, Tehran, Iran; ^4^Department of Nursing, Sabzevar Branch, Islamic Azad University, Sabzevar, Iran; ^5^Department of Nursing, Tabas Branch, Islamic Azad University, Tabas, Iran; ^6^Research Center for Health Management in Mass Gathering, Red Crescent Society of the Islamic Republic of Iran, Tehran, Iran; ^7^Center for Vaccine Development, University of Maryland School of Medicine, Baltimore, MD, United States; ^8^Mycobacteriology Research Center (MRC), NRITLD, Shahid Beheshti University of Medical Sciences, Tehran, Iran; ^9^Department of Naturopathic Medicine, Tehran, Iran; ^10^Priority Research Centre for Asthma and Respiratory Disease, Hunter Medical Research Institute, University of Newcastle, Newcastle, NSW, Australia

**Keywords:** ARDS, COVID-19, Borage, cytokine storm, CRP, TNF-α, IL-6

## Abstract

**Introduction:**

COVID-19 (coronavirus disease-2019) still causes a high rate of death globally with no definite curative treatment described. The traditional plant Borage (Borago officinalis L.) is a good source of gamma-linolenic (GLA). We hypothesized that Borage plus syrup (BPS) would be beneficial in severe COVID-19 patients within an intensive care unit (ICU) setting.

**Materials and methods:**

A pilot single center, randomized trial with no placebo was undertaken. A total of 60 PCR-positive severe COVID-19 participants admitted to ICU from June 2020–December 2020 at Masih Daneshvari Hospital Tehran-Iran gave informed consent. The participants were randomly assigned to either Borage Plus Syrup (BPS, 5 ml for 5 days) (*n* = 30) or standard care (IFN-β and favipiravir) as a control group (*n* = 30). Pao2/Fio2, serum ferritin, CRP, bilirubin, IL-6, TNF-α, ALT, AST, PCT and serum IL-8 was measured upon admission and on release.

**Results:**

All the measured parameters decreased significantly with BPS treatment. In the control group, most parameters significantly improved apart from AST and PCT. In addition, the suppression of serum TNF levels in the BPS group was greater than that seen in the control group (*P* ≤ 0.05). Moreover, the length of ICU stay was significantly lower in the BPS group compared with the control group (*P* ≤ 0.05).

**Conclusion:**

Our study shows that addition of BPS to the standard treatment regime of COVID-19 patients in ICU improved outcomes and reduced the length of ICU treatment. Natural products could be considered as new approaches for reducting the harmful consequences of COVID-19.

## Introduction

Coronavirus 2019 (COVID-19) is a pandemic that has increased mortality worldwide. According to the WHO statistics, as of 29 May 2022, over 526 million confirmed cases and over six million deaths have been reported globally leading to nearly 4,680,008 deaths (1). Iran was among the countries most affected by the pandemic with daily reports from the Health and Medical Education Ministry showing high numbers of infected subjects. By September 2021, nearly 5,378,408 Iranians were infected with COVID-19 leading to approximately 116,072 deaths (1).

The disease typically starts with mild symptoms resembling the common cold with clinical manifestations such as fever (in 98–88% of the cases), fatigue, dry cough, upper respiratory tract obstruction, dyspnea, sputum production, muscle pain, and gastrointestinal, blood lymphopenia, increased the prothrombin (PT) prolongation, elevated C reactive protein (CRP), and lactate dehydrogenase (LDH). Lung opacity, significantly reduced lymphocyte count, as well as the increased neutrophil count is observed in patients with severe disease. Serum levels of inflammatory cytokines including IL-6, G-CSF, IP-10, MCP1, MIP1A, and TNF are elevated in COVID-19 patients within intensive care units (ICU), which indicates the occurrence of cytokine storm in these patients (2–4).

The cytokine storm severely damages several organs, causes a deterioration in the patient’s health status, increases the ICU stay time, increases the patient’s dependence on mechanical ventilation and is associated with an increased mortality rate (3, 5). Considering the COVID-19 pandemic and the then absence of any specific treatments for the disease, many attempts were made to re-purpose different therapies in an effort to reduce the mortality rate and improve the clinical symptoms of the patients (3). Among the proposed treatments for controlling the cytokine storm in patients with COVID-19 is treatment with a high-dose corticosteroid (6). Corticosteroids induce severe immune suppression, which increases the risk of nosocomial infections, such as ventilator-associated pneumonia (VAP) which results in a raised mortality rate (6).

The use of complementary medicine and herbal medicine as safe and low-cost treatment approaches have, therefore, been considered (7). For example, AM3 supplement is a glycophosphopeptide derived from Ricinus communis protein and phosphorylated glucomannan polysaccharide from Candida utilis yeast. AM3 supplementation improves COVID-19 outcomes by decreasing the systemic levels of IL-6, TNF-α, CRP and ferritin and the enzymes ALT, AST, LDH, CK, and MB (8). Using medicinal plants for alleviating physical problems, e.g., inflammation is a custom in Iranian traditional medicine (9). Among the herbal candidates is an extract from borage–the Borage plus syrup (BPS) which was recently shown to alleviate inflammation in various *in vivo* and *in vitro* studies (10–13). For example, borage can alleviate of Alzheimer’s disease (AD)-induced cognitive dysfunction by halting the decline in hippocampal antioxidant status (10). Borage officinalis treatment also reduced the clinical symptoms of asthma including coughing, dyspnea, wheezing, nocturnal symptoms and airway hyperresponsiveness, but it was unable to reduce the inflammation that is a feature of asthma (11). The effects of borage and it’s metabolites extensively proposed by Das and co-workers (14–16). The Iranian Borage flower has also been used to treat infectious disorders and have antifebrile properties. Borage syrup was considered to be a treatment for jaundice, itch, and ringworm in addition to treating fever (12). Thus, in the current study we aimed to assess the effect of the BPS in severe COVID-19 patients in the ICU.

## Materials and methods

The present study is a randomized clinical trial that was conducted without a placebo control arm. All participants completed an informed consent form before enrolling in the study. The trial was approved by the Masih Daneshvari Ethical committee (IR.SBMU.NRITLD.REC.1399.059). The study population consisted of patients admitted to the COVID-19 ICU wards June 2020–December 2020 at Masih Daneshvari Hospital Tehran-Iran. A total of 60 patients were included in this study.

The inclusion criteria of the study included a confirmed COVID-19 diagnosis based on the clinical criteria agreed by a specialist, confirmed positive PCR, CT images of lungs, willingness for participation in the study and written informed consent. The exclusion criteria of this study was patient death or unwillingness to continue the study at any point for any reason. The participants were randomly divided into a control (*n* = 30) and an intervention group (*n* = 30). Serum levels of ferritin, CRP, bilirubin, IL-6, TNF-α, ALT, AST, PCT, IL-8 were measured in both control and it intervention groups at admission time and 5 days after treatment. All participants were treated according to the standard WHO protocol at the time with IFN-β and Favipiravir (600 mg three times a day) with patients in the intervention group receiving an additional 5 ml of BPS orally for 5 days. The dose of 5 ml for 5 days was selected based on the previous described effect on the cytokine storm (11, 17).

The sample size has been calculated by one of the key variables in this study, length of stay in ICU. Standard deviation of length of stay was estimated based on a pilot sample included 15 patients, (*S* = 2.5).

### Borage plus syrup preparation

Borage plus syrup is produced by the Iranian Maad Lotus Company (Tehran, Iran) and licensed by Iranian Health Ministry (number 11849). The fatty acid composition of the borage oil was linoleic acid (35–38%), oleic acid (16–20%), palmitic acid (10–11%), stearic acid (3.5–4.5%), eicosenoic acid (3.5–5.5%), and erucic acid (1.5–3.5%) (18). BPS syrup (5 ml) was given to patients at a dose of 100 mg/ml for 5 days which gave a GLA dose of 20 mg/ml (19).

### Blood sampling

Before the start of the trial and 5 days after the first treatment with Borage, venous blood samples were collected. The blood samples were centrifuged for 15 min at 380 × *g* to obtain serum. Serum samples were isolated and then stored at −20°C until analysis.

### Serum cytokine determination

A total of 3 ml whole blood without anticoagulant was harvested and after isolation of serum used to measure analytes using ELISA. The serum levels of TNF, IL-6, and IL-8 were measured using commercially available ELISA kits (DIASource, Belgium and eBioscience, USA) exactly according to the manufacturer’s instructions. Assays were read at 450 nM wavelength in an ELISA plate reader.

### Statistical analysis

The data was analyzed using SPSS version 22 (SPSS Inc., Chicago, IL, USA). A *p*-value of less than 0.05 was considered significant. Quantitative data were presented as mean ± SD and qualitative data were shown as a number (percentage). The normality of the quantitative variables was tested using the Kolmogorov–Smirnov test. To compare parametric variables between the two groups, a *t*-test and, if necessary, a non-parametric Mann–Whitney *U* test was used. Comparisons of before and after treatment was analyzed using a paired *t*-test for normal data and Wilcoxon’s test for non-parametric data. Relationships between categorical variables were performed using Cross Tabulation (Pearson Chi-Square Test).

## Results

**FIGURE 1:**
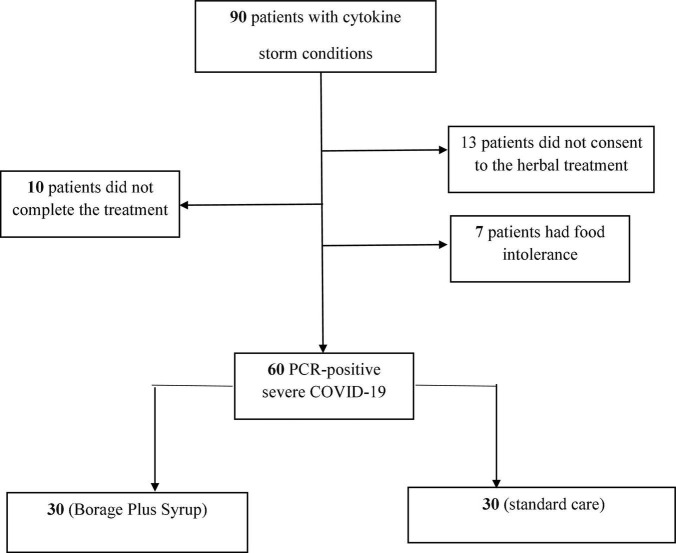
CONSORT flowchart.

The flowchart for patient recruitment shows the number of patients excluded from the study (30) and the number of subjects (60) included (Figure 1). The average age of the patients was 56.9 ± 13 years in the control group and 56.8 ± 12.3 years in the intervention (BPS) group. The mean APACHE score was 24.1 ± 2.8 in both groups. The average SOFA score in the intervention (9.43 ± 1.7) and control (9.46 ± 1.5) groups were also similar. A total of 14 patients (23.33%) of the 60 participants in this study received mechanical ventilation (MV), while 46 patients (76.67%) received non-invasive ventilation (NIV). Regarding underlying diseases; 13 patients (21.66%) suffered hypertension and 16 patients (26.66%) had diabetes mellitus whilst 4 patients (6.66%) suffered both diabetes mellitus and hypertension. A total of 8 patients (13.34%) suffered cardiovascular diseases with 5 patients (8.34%) suffering from diabetes mellitus, cardiovascular diseases and hypertension. Finally, 14 patients (23.34%) did not have any known underlying diseases (Table 1). None of these variables differed between the BPS and usual care study groups.

**TABLE 1:** Demographic information of participants.

	Groups		
	B and IFN-β and F (*n* = 30)	IFN-β and F (*n* = 30)	*P-value*
Sex (male *n*, %)^#^	21 (70.0%)	23 (76.7%)	0.55
Age (year) (mean ± SD)*	56.8 ± 12.3	56.9 ± 13.0	0.99
APACHE II (mean ± SD)*	24.1 ± 2.8	24.1 ± 2.8	1.00
SOFA (mean ± SD)*	9.43 ± 1.7	9.46 ± 1.5	0.93
Oxygen therapy, *n* (%)^#^			1.00
MV	7 (23.3)	7 (23.3)	
NIV	23 (76.7)	23 (76.7)	
Comorbidity, *n* (%)^#^			0.19
HTN	6 (20.0)	7 (23.3)	0.75
DM	10 (33.3)	6 (20.0)	0.24
HTN + DM	3 (10.0)	1 (3.3)	0.30
Cardiovascular	5 (16.7)	3 (10.0)	0.44
Cardiovascular + HTN + DM	3 (10.0)	2 (6.7)	0.64
None	3 (10.0)	11 (36.7)	0.01

*Comparisons between the groups were performed using Student’s *t*-test/Mann–Whitney *U* test.

^#^Relationships between categorical variables were analyzed using Cross Tabulation (Pearson Chi-Square Test).

A *p*-value of less than 0.05 was considered significant.

AT, after treatment; BT, before treatment; B, Borage; DM, Diabetes mellitus; F, Favipravir; IFN, Interferon; SOFA, sequential organ failure assessment score; NIV, non-invasive ventilation; MV, mechanical ventilation; HTN, hypertension.

### Cytokines and serum analytes

Table 2 shows the comparison of the serum level of cytokines between the BPS intervention and control groups before and after treatment. There were no significant differences in the levels of the analytes measures at baseline between the control and BPS treatment groups. The levels of all analytes were significantly suppressed in the BPS intervention group after treatment. The control group also saw significant reductions in most analytes over time except for AST and PCT. In addition, Pao2/Fio2 in both groups was significantly increased with treatment.

**TABLE 2:** Comparison outcome parameters between two participant groups.

Items	Groups				*P-value*			
	IFN-β and F and BPS (*n* = 30) Mean ± SD		IFN-β and F (*n* = 30) Mean ± SD		BPS and IFN-β and F (BT vs. AT)	IFN-β and F (BT vs. AT)	BPS& IFN-β and F (BT) vs. IFN-β and F (BT)	BPS and IFN-β and F (AT) vs. IFN-β and F (AT)
	BT	AT	BT	AT				
Pao2/Fio2	185 ± 52.6	238 ± 74.7	185 ± 52.9	239 ± 75.5	≤ 0.0001	≤ 0.0001	0.98	0.95
Ferritin	819 ± 292	460 ± 190	898 ± 278	538 ± 255	≤ 0.0001	≤ 0.0001	0.28	0.18
CRP	55.2 ± 17.8	34 ± 19.9	51.7 ± 13.0	26.5 ± 12.2	≤ 0.0001	≤ 0.0001	0.38	0.84
Bilirubin	52.1 ± 11.3	27.6 ± 8.0	53.6 ± 10.8	31.5 ± 9.8	≤ 0.0001	≤ 0.0001	0.58	0.10
IL-6	25.5 ± 9.8	16.1 ± 11.1	26.3 ± 9.8	14.8 ± 6.8	≤ 0.001	≤ 0.0001	0.74	0.61
TNF-α	16.7 ± 7.2	6.5 ± 2.3	16.8 ± 7.0	10.9 ± 3.2	≤ 0.0001	≤ 0.0001	0.92	≤ 0.0001
ALT	38.1 ± 12.7	34.7 ± 17.3	42.3 ± 15.6	35 ± 19.9	0.038	0.001	0.26	0.94
AST	33.5 ± 12.4	25.5 ± 13.8	35.3 ± 8.5	28.4 ± 18.8	0.001	0.055	0.51	0.50
PCT	0.33 ± 0.23	0.20 ± 0.17	0.27 ± 0.20	0.21 ± 0.17	≤ 0.001	0.174	0.25	0.87
IL-8	50.0 ± 8.5	17.9 ± 8.4	51.0 ± 8.8	20.2 ± 9.6	≤ 0.0001	≤ 0.0001	0.67	0.33
Length of stay in ICU (days)	8.2 ± 1.9		9.3 ± 2.2		≤ 0.045			
Mortality rate n (death %)	7 (23.3)		7 (23.3)					

Comparisons between the groups were performed using Student’s *t*-test/Mann–Whitney *U* test. Comparisons of before and after treatment was analyzed using a paired *t*-test/Wilcoxon.

*p*-value of less than 0.05 was considered significant.

AST, aspartate aminotransferase; ALT, alanine aminotransferase; AT, after treatment; BT, before treatment; BPS, Borage; CRP, C reactive protein; F, Favipravir; IFN, Interferon.

Importantly, the reduction in TNF expression was significantly higher in the BPS intervention group than in the control group (*P* ≤ 0.0001). Moreover, the length of ICU stay was significantly lower in the intervention group compared with the control group (Table 2).

## Discussion

In current study we applied the herbal extract BPS to severe COVID-19 patients within ICU as an add-on therapy to standard treatment. We show that the Pao2/Fio2 in intervention and control groups is increased however serum ferritin, CRP, bilirubin, IL-6, IL-8, TNF-α, ALT, AST, and PCT were decreased in the BPS intervention group. Similar effects on cytokine levels were seen with standard care although no significant attenuation of AST or PCT levels was observed. Interestingly, the reduction in TNF levels was significantly greater in the BPS intervention group. There was a small but significant reduction in the length of ICU stay in the BPS treatment group compared to the standard intervention group.

So far, no effective antiviral treatment is available to treatment of COVID-19 disease and many treatment approaches are geared toward reducing the inflammatory response and to preventing severe lung damage. BPS contains a mixture of medicinal plants such as Borage, lemongrass, yarrow, chamomile, and chicory, all of which have confirmed anti-inflammatory effects in traditional medicine (20). Hashemian et al. showed that BPS can effectively reduce the level of inflammatory cytokines in ARDS patients (17). This resulted in diminishing the duration of mechanical ventilation (MV) as well as disease morbidity and mortality in patients with ARDS (20).

Borage contains several anti-inflammatory and anti-oxidant components (21, 22). This is proposed to account for the beneficial effects seen including anxiolytic, sedative, anti-inflammatory and analgesic actions against the common cold (19). In addition, to antioxidant effects, BPS can stimulate lymphocyte proliferation and antibacterial actions against staphylococcus aureus for example (23). Borage, containing mainly borage extract with the addition of mild hyssop and mint extract, GLA and eicosapentaenoic acid (EPA), GLA and EPA reduce the levels of inflammatory cytokines and chemotactic factors in bronchial alveolar fluid (BAL) of ARDS patients (24). GLA administered in the form of borage oil gets metabolized to dihomo-γ-linolenic acid (DHLA), arachidonic acid (AA) and prostaglandine E2 (PGE1) and other eicosanoids (25). Its GLA content has the capacity to inhibit TNF-α (11). There are no published direct effects of GLA/LA on the SARS-CoV-2 demonstrated using *in vitro* studies. However, polyunsaturated ω-3 fatty acids can inhibit ACE2-SARS-CoV-2 binding and cellular entry (26).

Furthermore, Borage reduces the mortality rate, duration of mechanical ventilation and the insufficiency of vital organs in patients with ARDS (27).

The fatty acids found in Borago officinalis and its Iranian species Echium amoenum include palmitic, linoleic, stearic, and linolenic acids. This plant’s flower has a long history of use in folk medicine as a bronchodilator (11) but borage oil can reduce the production of ROS, eliminates free radicals, and lessens the impact of inflammatory proteins (10). BPS has been prescribed at a dose of 1.4–2.8 g/day for arthritis and other inflammatory diseases in clinical trials (28). For example, borage oil was clinically effective in children with atopic dermatitis in a double-blind, placebo-controlled clinical experiment. In particular, transepidermal water loss (TEWL) on the dorsal skin was significantly reduced in the borage-treatment group. Importantly, patients had no negative effects (29). Furthermore, borage oil containing γ-linoleic acid decreased inflammatory and non-inflammatory acne lesions as well as lesional IL-8 levels with no serious adverse effects being recorded (30).

The effects of boragina on bone mineral density (BMD) may result from greater GLA concentrations. In fact, the cyclooxygenases that transform GLA into PGE1, which has anti-inflammatory properties that may help bone and recover BMD. This theory is supported by the finding that, in the SAMP8 mouse strain (a progeria model), spleen weight and CRP levels were associated with a reduction in IL-6 and IL-6R expression in bone tissues as compared to a wild type control strain (31). Steatohepatitis and cardiovascular illnesses both exhibit high levels of oxidative stress, inflammation, and lipid imbalance and may be good targets for borage treatment supporting data in rat models (32).

In the current study, the reduced level of TNF-α indicates an anti-inflammatory effect of BPS in COVID-19 disease. We speculate, therefore, that this herbal syrup given in the early stages of COVID-19 may improve the condition of patients and reduce the risk of a cytokine storm developing, hospitalization and thereby reduce the mortality rate. Corticosteroids are a good supportive treatment strategy for patients with severe COVID-19 (33) but are associated with several side effects including immune system suppression and delayed healing. The advantages of BPS could be considered as a natural and safe herbal products with very low side effects.

Dynamic abnormalities in liver function parameters are common in COVID-19 patients, and associated with disease severity and mortality of disease (34). Elevated liver enzymes revealed in 15–58% of COVID-19 patients (35). The most common patterns of liver enzyme abnormalities in patients with COVID-19 include elevated AST and ALT (35). In the current study we show that patients who were treated with BPS had reduced serum levels of AST. Previous studies showed that procalcitonin (PCT) levels are increased in severe COVID-19 patients and that this may be considered as a prognostic factor in the severity of COVID-19 disease (36). In the current study we demonstrate that BPS reduced both AST and PCT levels in contrast to the lack of effect in the standard care group. This may contribute to the beneficial effects of BPS in this ICU cohort of COVID-19 patients.

One limitation of current study is small size of the study conducted in a single center. Furthermore, we did not include a placebo group. However, we show that treatment with BPS is able to decrease PCT and TNF-α more than in the standard care control group and reduce the duration of ICU stay. In addition, although APACHE scores were similar between groups there were significantly fewer subjects with no comorbidities in the borage group. The results of the present study show significant effects of combined treatment of herbal and conventional medications on the treatment effectiveness and alleviation of COVID-19 disease symptoms. This suggests the potential role of herbal products such as BPS in the treatment of patients with COVID-19 or ARDS. Considering that there is still no known treatment for the inflammatory phase of COVID-19, the use of safe herbal remedies along with medical treatments will reduce mortality and increase the effectiveness of therapeutic drugs. In addition, it is possible that the efficacy of borage oil is due to GLA alone and future studies should investigate this. Examination of plasma or serum fatty acid components in future studies may help resolve this and indicate correlations with the effects of borage oil on cytokine levels.

Further studies are needed to characterize the effectiveness of BPS in the early stages of COVID-19 particularly in people who remain resistant to being vaccinated. The precise molecular effects of BPS also warrant additional research.

## Data availability statement

The original contributions presented in this study are included in the article/supplementary material, further inquiries can be directed to the corresponding author.

## Ethics statement

The studies involving human participants were reviewed and approved by the Shahid Beheshti Medical University. The patients/participants provided their written informed consent to participate in this study.

## Author contributions

SH desinged the proposal. NS, SZ, HJ, MH, MJ, RE, BK, and MF recruited patients and DIS experiments and approved last version of manuscript. ND analyzed statistical methods. MF, IMA, and EM revised last version of manuscript. All authors contributed to the article and approved the submitted version.
